# Green to red photoconversion of GFP for protein tracking *in vivo*

**DOI:** 10.1038/srep11771

**Published:** 2015-07-07

**Authors:** Amirali Sattarzadeh, Reza Saberianfar, Warren R. Zipfel, Rima Menassa, Maureen R. Hanson

**Affiliations:** 1 Cornell University, Department of Molecular Biology and Genetics, Ithaca, NY, 14853 USA; 2 University of Western Ontario, Department of Biology, London, ON, N6A 5B7 Canada; 3 Cornell University, Department of Biomedical Engineering, Ithaca, NY, 14853 USA; 4 Agriculture and Agri-Food Canada, London, ON, N5V 4T3 Canada

## Abstract

A variety of fluorescent proteins have been identified that undergo shifts in spectral emission properties over time or once they are irradiated by ultraviolet or blue light. Such proteins are finding application in following the dynamics of particular proteins or labelled organelles within the cell. However, before genes encoding these fluorescent proteins were available, many proteins have already been labelled with GFP in transgenic cells; a number of model organisms feature collections of GFP-tagged lines and organisms. Here we describe a fast, localized and non-invasive method for GFP photoconversion from green to red. We demonstrate its use in transgenic plant, Drosophila and mammalian cells *in vivo*. While genes encoding fluorescent proteins specifically designed for photoconversion will usually be advantageous when creating new transgenic lines, our method for photoconversion of GFP allows the use of existing GFP-tagged transgenic lines for studies of dynamic processes in living cells.

Fusions of Green Fluorescent Protein (GFP) and its derivatives are extremely valuable tools for examining gene expression, protein and RNA localization, protein-protein interactions, protein synthesis and degradation, and organelle movement. The development of photoconvertible fluorescent proteins such as mEosFP, tdEosFP, Dendra, Dronpa, Kaede, KikGR, mOrange allows new questions to be asked concerning dynamic processes in living cells^1^,^2^,^3^,^4^,^5^,^6^. However, a number of collections of GFP-tagged organisms had been made before these proteins became available for use. For example, a collection of strains (http://yeastgfp.yeastgenome.org/) expressing full-length GFP-tagged proteins is available for yeast^7^,^8^. In Drosophila, there is a collection (http://cooley.medicine.yale.edu/flytrap/) in which GFP has tagged full-length endogenous proteins expressed from their endogenous loci or used in an enhancer trap^9^,^10^. In Arabidopsis, lines are available with GFP tags on transcription factors^11^, with GFP as promoter-reporters^11^, and with GFP labels on subcellular structures^12^. In addition to specific collections, many investigators with interests in particular proteins or subcellular locations have produced cell lines or organisms with GFP tags. For example, various transgenic mouse lines (http://www.jaxmice.jax.org/) have been produced with GFP labels on proteins^13^; retransforming such lines with genes encoding new photoconvertible proteins would require considerable time and expense. Because many proteins have already been tagged with EGFP or GFP modified by an S65T mutation, being able to photoconvert these common forms of GFP would allow the use of existing transgenic lines for studies of dynamic changes.

EGFP has been known to change color after 488 nm illumination under low oxygen conditions^14^, low oxygen with added FAD^15^ or by added oxidizing agents such as potassium ferricyanide, benzylquinone (BQ) or MTT^16^. The fluorescence spectra of the photoconverted species varied depending on the conditions used to catalyze the conversion. The “red” emission maximum was at 560 nm in the FAD-mediated conversion^15^, while three peaks were reported (560, 590 and 600 nm) under anaerobic conditions (no added FAD)^14^. The addition of electron acceptors such as BQ resulted in 600 nm emissions with the exception of one report of a 675 nm peak^17^. Performing GFP photoconversion in low oxygen requires placing the tissue in special conditions, and use of oxidizers obviates one of the advantages of GFP—the ability to image without concerns about uptake of additional chemicals. We report here a method that can be used to convert GFP from green to red without a special environment or exogenous chemicals. We show its effectiveness in plant, mammalian, and Drosophila cells.

## Results

### Several common variants of GFP can be photoconverted

**Figure 1:**
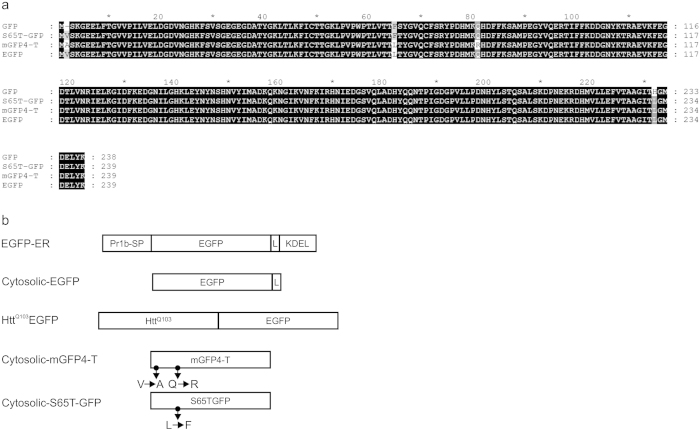
GFP variants used for photoconversion. (**a**) Alignment of the coding region of the GFP variants. The alignment was created using GeneDoc (http://www.nrbsc.org/gfx/genedoc/). (**b**) Schematic representation of GFP transgenes used in photoconversion experiments. Pr1b-SP, secretory signal peptide from the tobacco pathogenesis-related protein 1; EGFP, enhanced green fluorescent protein; L, linker (GGGS)_3_; KDEL, endoplasmic reticulum retrieval signal peptide; Htt^Q103^, exon 1 of human Htt^Q103^ gene; Cytosolic mGFP4-T in tobacco cell culture and cytosolic S65T-GFP in Drosophila gut cells. In cytosolic mGFP4-T, V (valine) was replaced by A (alanine), and Q (glutamine) was replaced by R (arginine) relative to EGFP. In cytosolic S65T-GFP, L (leucine) was replaced by F (phenylalanine) relative to EGFP.

While performing photobleaching studies on plant cells labeled with GFP, we made the unexpected observation that EGFP, mGFP4-T, and GFP modified by an S65T mutation can be photoconverted by 405 nm light *in vivo*. The sequences of the variant GFPs we have analyzed are shown in Fig. 1a.

**Figure 2:**
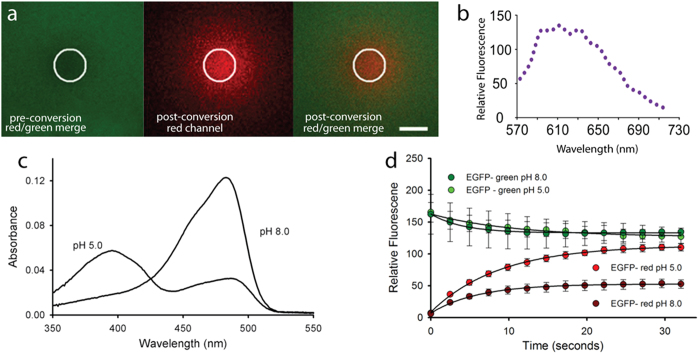
Photoconversion of purified EGFP *in vitro*. (**a**) Typical pre- and post-photoconversion images of a thin (~10 μm) layer of 30 μM EGFP using 405 nm excitation delivered through a 1.2 NA objective to the region within the circle using the Zen software bleaching mode (Green channel: 505–525 nm; Red channel: 580–670 nm; scale bar: 50 μm). See also Supplementary Movie S1. 10 iterations of 257 ms each were performed. (**b**) Emission spectra of photoconverted red state EGFP acquired using the Zeiss 710 spectral detector (5 nm bandwidth intervals). (**c**) Absorption spectra of EGFP (~2 μM) at pH 5.0 and pH 8.0. (**d**) Time course of the increase in red fluorescence with successive 405 nm irradiations within the region of interest (ROI). EGFP (30 mM) was subjected to a sequence of 10 “bleach” iterations over the ROI (1.8 seconds) followed by image acquisition using 488 and 561 nm illumination. The 405 nm power at the sample was at 2.5 mW, corresponding to 6.8 MW/cm^2^ at the focus of the 1.2 NA objective lens. Plotted data points are the average pixel values across the entire field of view which estimates the total photo-product produced. Error bars represent the SEM of three trials at pH 5.0 and two at pH 8.0. Black lines are fits to exponential increase for the red channel (1-exp(-αt)) and decrease (exp(-αt)) for the green. Returned values of α were ~0.14 s^−1^ for all data sets except the decay of the pH 8.0 green channel (which was 0.24 s^−1^).

To verify whether EGFP could be photoconverted *in vitro* from its normal green state to a red state, we investigated irradiation of purified EGFP. By using the Zeiss 710 bleaching mode with a 405 nm laser for photoconversion, rather than 488 nm as used in the previous reports, purified EGFP was imaged before and after application of the 405 nm light. The green-state of purified EGFP was imaged with excitation at 488 nm, and the emission was acquired between 505–525 nm. The photoconverted EGFP (red state) was monitored using 561 nm excitation and detected between 580–670 nm. Photoconversion occurred, as demonstrated by the decrease in green fluorescence and the simultaneous increase in red fluorescence (Fig. 2a). We observed a maximum emission peak around 612 nm when using 561 nm excitation (Fig. 2b). The red species of EGFP is not excited by 488 nm light and we could detect no red species formed using 488 nm light alone for photoconversion.

The need for 405 nm excitation indicated that we were most likely photoconverting the protonated or “A” form of the chromophore^18^ as indicated by the increased yield of red photo-product at pH 5.0 compared to pH 8.0 under identical illumination conditions and total EGFP concentration (Fig. 2d). At pH 5.0, the A_405_ of EGFP is ~2.6 times higher than at pH 8.0 (Fig. 2c). This approximately accounts for the increase in red photo-product as shown in the time course data (Fig. 2d), where a 2.3 fold difference in red fluorescence was found between pH 5.0 and pH 8.0. The rate of red species formation was the same at both pH values (~0.14 s^−1^ under our conditions) indicating that the protonated form is being converted and it is the concentration of the “A” form that matters. This finding also indicates that cellular compartments with higher pH may be less suitable for imaging of photoconverted GFP unless concentrations are relatively high. The photoconversion appears to be irreversible and red-GFP was still visible several hours after photoconversion (data not shown) in our *in vitro* experiments.

**Figure 3:**
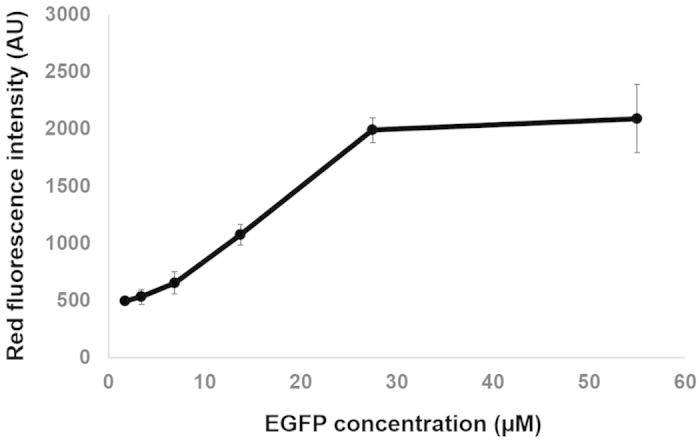
Correlation between EGFP concentration and red fluorescence intensity following irradiation. EGFP was diluted in PBS buffer at the following concentrations: 0.875, 1.7, 3.4, 6.8, 13.75, 27.5, 55 μM. All photoconversion and imaging parameters were kept similar for all tested EGFP concentrations. Data are from 3 independent experiments.

GFP will be present in a variety of concentrations in transgenic cells and organisms, depending on levels of protein expression and stability. We investigated the intensity of the fluorescent signal relative to concentration of purified GFP. Sufficient GFP must be present to observe the photoconverted form, and the signal appears to saturate at approximately 30 μM (Fig. 3).

### GFP variants can be photoconverted *in vivo*

**Figure 4:**
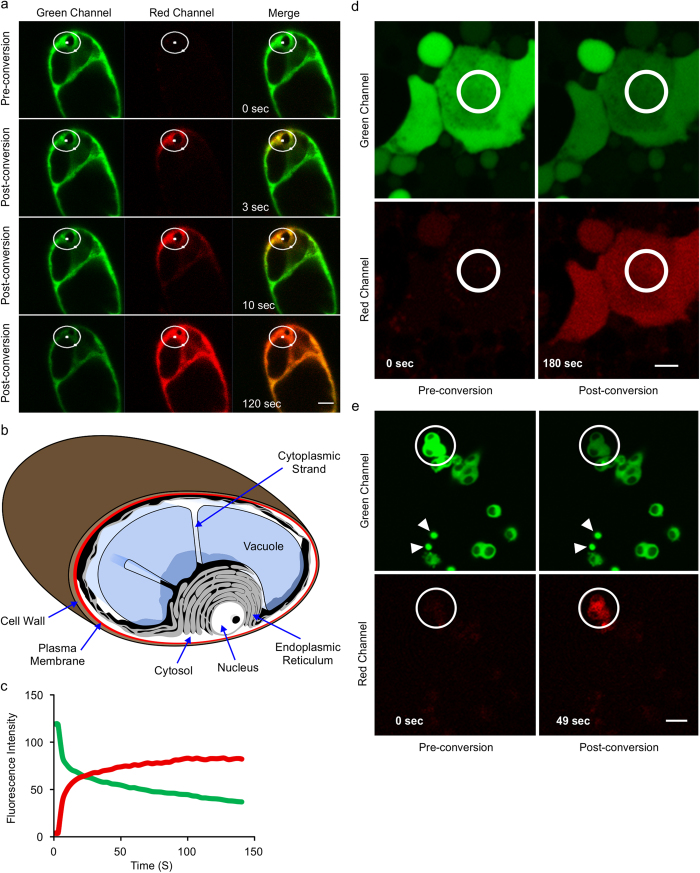
Photoconversion of GFP from green to red state *in vivo*. Single snapshots from the pre-photoconversion and post-photoconversion are shown. (**a**) Tobacco suspension cell culture in which GFP is targeted to cytosol. Photoconverted GFP spreads through the cytosol via cytoplasmic strands. Photoconversion was performed at 50% laser power and 30 iterations of 111 milliseconds (ms). (**b**) Schematic representation of a tobacco suspension culture cell. The cell wall (brown) surrounds the plasma membrane (red). A large vacuole (blue) occupies the majority of the space inside the cell. The endoplasmic reticulum network (gray) surrounds the vacuole and the nucleus (white) and extends through the rest of the cytosol (white). Cytoplasmic strands exist as extensions of the cytosol between organelles. (**c**) Changes in fluorescence intensity in the irradiated area in (**a**) over time indicate increasing red and decreasing green fluorescence. (**d**) Drosophila gut cells from 3rd instar larvae expressing S65T-GFP. (**e**) Rat PC12 cells expressing EGFP fusion to exon 1 of human Htt^Q103^ gene. White arrowheads highlight cytosolic inclusions. Images acquired by 30 iterations with the duration of 190 ms each. Laser power was adjusted at 70%. Bars 10 μm.

To determine whether GFP could be converted from the green to the red state *in vivo*, we performed photoconversion experiments in transgenic tobacco cells in which GFP was localized in the cytosol^19^ (Fig. 4a-c). Converting GFP from green to red state resulted in rapid diffusion of the red form within the plant cytosol. In less than 3 seconds after irradiation, red-GFP could be detected in the red channel. The photoconverted red-GFP then diffused through the whole cell and was readily visible throughout the cell within 2 minutes (Fig. 4a and Supplementary Movie S2). Fluorescence intensity was measured in the area indicated by a white circle in the irradiated area. There is a noticeable decrease in intensity of the green signal and a concomitant increase in intensity of the red signal, which is an indication of photoconversion from green to red state (Fig. 4c). We detected no red signal when plant cells not expressing GFP were irradiated and imaged under the same conditions.

We could also observe the photoconversion of EGFP in other eukaryotic model organisms, including Drosophila and rat cells. We obtained a Drosophila line in which S65T-GFP was expressed in gut cells from 3rd instar larvae^20^ (Fig. 4d), and a rat PC12 cell line expressing EGFP fused to exon 1 of the human Huntington poly Q (Htt^Q103^) gene^21^ (Fig. 4e). When Drosophila or rat cells were irradiated using conditions similar to those that were effective for plant cells, we observed photoconversion from the green to red state (Fig. 4d,e).

**Figure 5:**
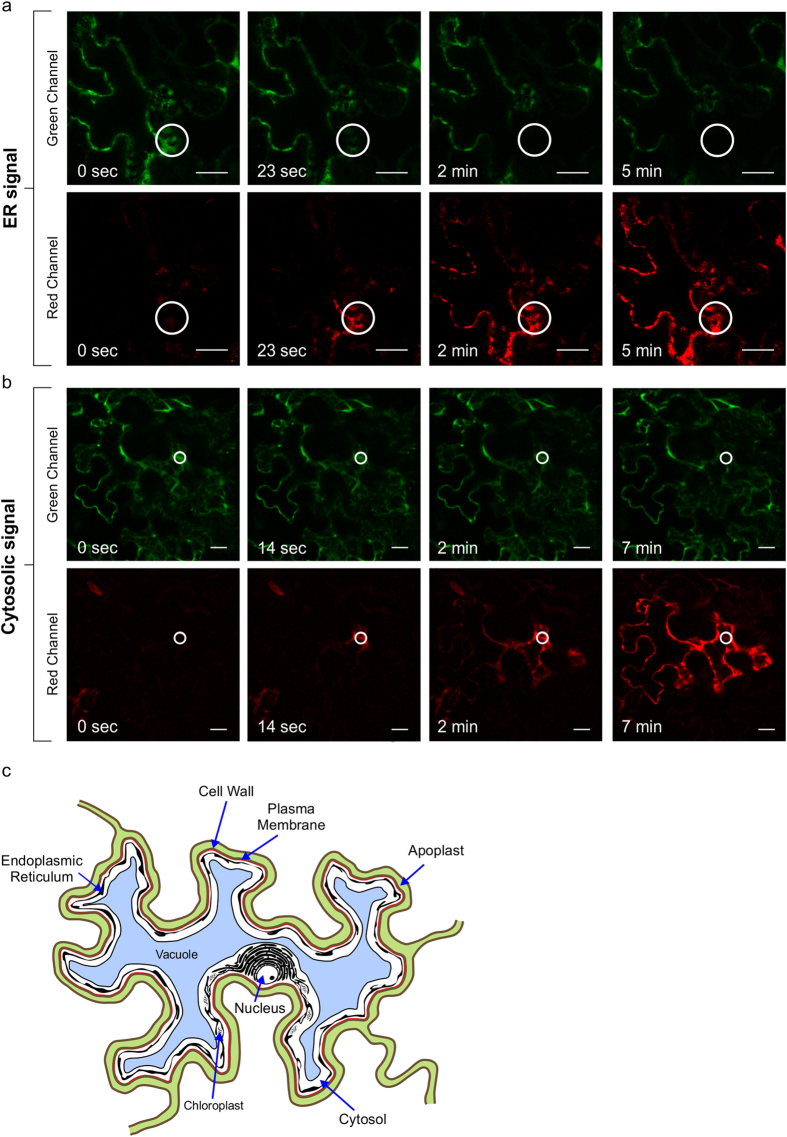
EGFP photoconversion occurs in *N. benthamiana* cells transiently expressing EGFP. (**a**) ER-targeted EGFP photoconverts from green to red upon excitation with 405-nm laser at 60% of laser power and 100 iterations with duration of 145 milliseconds each. The photoconverted red protein travels within the ER network of the cell. (**b**) Cytosolic EGFP photoconverts and spreads within the cytosolic space of the cell. Photoconversion was performed at 70% of laser power and 30 iterations with duration of 190 milliseconds each. White circles represent the irradiated area. Bar 20 μm. (**c**) Schematic representation of a *Nicotiana benthamiana* epidermal leaf cell. Puzzle-shaped epidermal cells are surrounded with the cell wall (brown). The space between the cell walls of two neighboring cells, apoplast, is shown in green. The plasma membrane (red) surrounds the cell. A large vacuole (blue) occupies the majority of the inner space of the cell. Endoplasmic reticulum (black) is localized around the nucleus and spreads throughout the cytosol (white), which is pushed against the plasma membrane by the vacuole. Chloroplasts are drawn in black as oval structures in the cytosol.

Transient expression methods are also frequently used to study the subcellular localization of GFP-tagged proteins. In plants, several methods exist for transient expression, including particle bombardment, infiltration of *Agrobacterium tumefaciens* (Agroinfiltration), or protoplast transfection. In addition to photoconversion of stably transformed tobacco cells, we were able to photoconvert EGFP that was transiently expressed in *Nicotiana benthamiana* epidermal leaf cells by Agroinfiltration (Fig. 5, Supplementary Movies S3-4). We were able to label the endoplasmic reticulum (ER) with GFP targeted to the ER and observe movement through the ER (Fig. 5a). Also, GFP located in the cytosol could be photoconverted and we could follow its movement through the cell (Fig. 5b).

## Discussion

Variant fluorescent proteins that have been designed for photoconversion applications will usually be superior to GFP in terms of rapidity of shift in emission and magnitude and may be brighter at low concentration. Thus, the primary advantage of using GFP in photoconversion is the ability to use existing transgenic lines.

Nevertheless, GFP has other advantages over the use of some other fluorescent proteins. Non-GFP photoconvertible fluorescent proteins sometimes exhibit unexpected subcellular localization behavior. One example is a study performed by Schattat *et al.*^22^, in which mEOS2 was used to assess movement of protein into plastid extensions termed stromules^23^. In contrast to prior and subsequent studies using GFP^24^,^25^,^26^, following photoconversion, mEOS2 did not move between plastid bodies and appeared to become “stuck” within the plastid body or partly into the stromule^22^. Another example of abnormal localization and loss of movement was observed in experiments with photoconvertible fluorescent protein fusions to the SHORT-ROOT (SHR) protein, which normally moves between different cell types in the root^27^. When tdEosFP was fused to SHR, the fusion protein exhibited abnormal intracellular localization and did not move into the endodermis of the root, unlike GFP fusions^28^.

Another benefit of GFP-based photoconversion is the absence of any random or auto-photoconversion in our tested samples. For some of the non-GFP-based photoconverting proteins, auto-photoconversion has been reported. For example, in mEosFP-cytosolic plants grown in bright fluorescent white light, up to a quarter of hypocotyl epidermal cells contained red nuclei, and in cells transiently expressing cytosolic mEosFP, auto-conversion was observed without any intentional photoconversion^8^. Another concern involved with the use of mEosFP is the partial photoconversion under short exposure times. Photoconversion of mEosFP happens in a concentric manner, therefore producing variability in shades, which is problematic since both partial photoconversion and co-localization of the green and red will produce yellow hues^29^.

Photoconversion of GFP also has an advantage in material labelled with multiple fluorophores. GFP photoconversion would avoid crosstalk between multiple fluorophores since its maximum emission peak at 612 nm is different from that of the most commonly used photoconvertible proteins such as mEOS2 (red) at 584 nm and Dendra2 (red) at 573 nm, or with other fluorescent proteins such as EBFP, ECFP, EYFP with maximum emission peaks at 445, 476, 527 respectively, and many other fluorescent proteins.

In cells with high expression levels of GFP, lower laser powers (20–30% corresponding to ~500 μW) and shorter exposure times can be used for photoconversion experiments. In cells with lower GFP expression, sequential and simultaneous imaging of cells during the photoconversion is recommended in order to prevent photodamage in the cells under irradiation. To track the *in vivo* movement of proteins, multiple irradiations with time intervals (from seconds to minutes) were found to be useful to follow trafficking of the photoconverted protein and photoconversion of newly synthesized/trafficked proteins in the irradiated area (Supplementary Movies 3-4).

In summary, we have demonstrated that GFP photoconversion can be added to the cell biologist’s toolkit for monitoring protein dynamics in transgenic cells, provided adequate amounts of GFP are expressed for sufficient sensitivity. Using GFP photoconversion is particularly advantageous when transgenic cells and lines have already been produced with GFP labelling, as re-transformation with a different gene encoding a new photoconvertible protein may not be necessary. The method we describe could find applications in a broad range of biological systems.

## Methods

### Constructs and transgenes

ER-EGFP and cytosolic-EGFP plant expression vectors and PC12 cell line expressing Htt^Q103^-EGFP were previously published^21^,^30^. The genotype of the Drosophila larvae is FM7i, P{w[+mC] = ActGFP}JMR3^20^.

### Strains used and culture conditions

For transient expression experiments, we used 7-week-old *N. benthamiana* plants grown at 22 °C with a 16 hour photoperiod at 110 μmol m^−2^ s^−1^. Plants were fertilized with the water soluble fertilizer (N:P:K = 20:8:20) (Plant Products, Brampton, ON, Canada) at 0.25 g/L. *A. tumefaciens* strain EHA105 containing the cytosolic-EGFP and ER-EGFP were grown to an optical density at 600 nm (OD_600_) of 0.5–0.8, and collected by centrifugation at 1000 ***g*** for 30 minutes. The collected pellets were resuspended in Agroinfiltration solution (3.2 g/L Gamborg’s B5 medium and vitamins, 20 g/L sucrose, 10 mM MES pH 5.6, 200 μM 4′-Hydroxy-3′, 5′-dimethoxyacetophenone) to a final OD_600_ of 0.30, and incubated at room temperature (21 °C) with gentle agitation for 1 hour. The suspension was then used for infiltration of the abaxial leaf epidermis of *N. benthamiana* with a 1 ml syringe (Conley *et al.*^30^)

Rat PC12 cells were grown in Dulbecco’s modified Eagle’s medium (DMEM) and supplemented with 10% FBS and 1% penicillin/streptomycin at 37 °C in 5% CO_2_. To induce the expression of Htt^Q103^-EGFP, cells were treated with 2.5 μM tebufenocide (Sigma, St. Louis, MI) for 24 to 48 hours. Tobacco cell suspensions were grown in NT1 medium^23^ with regular shaking at 22 °C. Drosophila larvae were grown at room temperature in standard corn meal agar^9^.

Purified EGFP was obtained by HisPur Ni NTA bead-based chromatography of the 6xHis-EGFP plasmid (Addgene, Cambridge, MA, USA) expressed in *E. coli*.

### Microscopy and imaging

A Zeiss LSM 710 confocal microscope equipped with a 10X dry, 40X or 63X water immersion objective was used for the imaging and photoconversion of GFPs. Green fluorescence was detected with a 505–530 nm band pass filter, and red fluorescent emission was detected with a spectral detector between 580–640 nm. EGFP fluorescence and absorption at pH 5.0 and pH 8.0 was measured in a MOPS-citrate buffer (120 mM KCl, 5 mM NaCl, 1 mM MgCl, 10 mM MES, 10 mM MOPS and 10 mM citrate) adjusted to the desired pH.

## Additional Information

**How to cite this article**: Sattarzadeh, A. *et al.* Green to red photoconversion of GFP for protein tracking *in vivo*. *Sci. Rep.* **5**, 11771; doi: 10.1038/srep11771 (2015).

## Supplementary Material

Supplementary Movies

Supplementary Movie S1

Supplementary Movie S2

Supplementary Movie S3

Supplementary Movie S4
