# Supplementary material for: Green to red photoconversion of GFP for protein tracking in vivo
Source: Sci Rep. 2015 Jul 7;5:11771. doi: 10.1038/srep11771 (PMC4493561; doi:10.1038/srep11771)
Supplement: Supplementary Movies [file srep11771-s1.pdf]

SUPPLEMENTARY INFORMATION

Green to red photoconversion of GFP for protein tracking *in vivo*

Amirali Sattarzadeh[1]*, Reza Saberianfar[2,4]*, Warren R. Zipfel[3], Rima Menassa[2,4] and Maureen R. Hanson[1]

[1]Cornell University, Department of Molecular Biology and Genetics, Ithaca, NY, USA

[2]University of Western Ontario, Department of Biology, London, ON, Canada

[3]Cornell University, Department of Biomedical Engineering, Ithaca, NY, USA

[4]Agriculture and Agri-Food Canada, London, ON, Canada

*These authors contributed equally to this work.

Supplementary Movie 1. Time-lapse series demonstrating photoconversion of purified EGFP. (Also see Fig. 1).

Supplementary Movie 2. Tobacco suspension cell culture labeled with S65T GFP in the cytoplasm undergoing photoconversion from the green state to the red state. (Also see Fig. 2a).

Supplementary Movie 3. Time-lapse confocal imaging of cells expressing ER targeted EGFP in *N. benthamiana* was performed to demonstrate the photoconversion and trafficking of EGFP in the ER network. The ROI (shown with white circles) was irradiated with 405 at 60% of laser power and 100 iterations every 30 seconds.

Supplementary Movie 4. Time-lapse confocal imaging of cells expressing cytosolic EGFP in *N. benthamiana* was performed to demonstrate the photoconversion and trafficking of EGFP in the cytosolic space of the cell. The ROI (shown with white circles) was irradiated with 405 at 70% of laser power and 30 iterations every 22 seconds.
